# Development of a marker-free mutagenesis system using CRISPR-Cas9 in the pathogenic mould *Aspergillus fumigatus*

**DOI:** 10.1016/j.fgb.2020.103479

**Published:** 2020-12

**Authors:** Norman van Rhijn, Takanori Furukawa, Can Zhao, Bethany L. McCann, Elaine Bignell, Michael J. Bromley

**Affiliations:** Manchester Fungal Infection Group, Division of Infection, Immunity and Respiratory Medicine, Faculty of Biology, Medicine and Health, University of Manchester, CTF Building, 46 Grafton Street, Manchester M13 9NT, UK; Lydia Becker Institute of Immunology and Inflammation, Manchester Collaborative Centre for Inflammation Research, Division of Infection, Immunity and Respiratory Medicine, Faculty of Biology, Medicine and Health, University of Manchester, Manchester Academic Health Science Centre, Manchester, UK

**Keywords:** CRISPR-Cas9, *Aspergillus fumigatus*, Marker-free, Mutagenesis, Selection-free

## Abstract

*Aspergillus fumigatus* is a saprophytic fungal pathogen that is the cause of more than 300,000 life-threatening infections annually. Our understanding of pathogenesis and factors contributing to disease progression are limited. Development of rapid and versatile gene editing methodologies for *A. fumigatus* is essential. CRISPR-Cas9 mediated transformation has been widely used as a novel genome editing tool and has been used for a variety of editing techniques, such as protein tagging, gene deletions and site-directed mutagenesis in *A. fumigatus*. However, successful genome editing relies on time consuming, multi-step cloning procedures paired with the use of selection markers, which can result in a metabolic burden for the host and/or unintended transcriptional modifications at the site of integration. We have used an *in vitro* CRISPR-Cas9 assembly methodology to perform selection-free genome editing, including epitope tagging of proteins and site-directed mutagenesis. The repair template used during this transformation use 50 bp micro-homology arms and can be generated with a single PCR reaction or by purchasing synthesised single stranded oligonucleotides, decreasing the time required for complex construct synthesis.

## Highlights

• *In vitro* assembled CRISPR/Cas9-gRNA complexes can mediate selection free transformation.
• Single gRNAs can be used to target mutagenesis effectively.
• Targeted point mutations can be achieved utilising single strand DNA oligonucleotides as repair template.

## 1 Introduction

*A. fumigatus* is a saprophytic fungus and the primary aetiological agent of invasive aspergillosis. This disease primarily affects immunocompromised individuals and causes over 300,000 life-threatening invasive infections annually. *A. fumigatus* also causes chronic and allergic diseases in immunocompetent individuals which affects around 3 million and 20 million people, respectively (Bongomin et al. 2017). Our understanding of the pathogen and host factors that contribute to these diseases is limited. Improvements in methods that facilitate genetic modifications in filamentous fungi are required to aid research in this area.

Unlike the model yeast *Saccharomyces cerevisiae*, targeted allele replacement in *A. fumigatus* is complicated by low rates of homologous recombination and the fact that replacement cassettes require long homology arms (LHA) of c. 1 kb (Stafa et al. 2017). Improvement in the frequency of homologous recombination can be achieved by employing strains deficient in the non-homologous end-joining (NHEJ) pathway (Δ*ku80*, Δ*ku70, and* Δ*lig4* strains) (da Silva Ferreira et al., 2006, Ishibashi et al., 2006). However, LHAs are still required to facilitate homologous recombination in these strains (da Silva Ferreira et al. 2006). Construction of transformation cassettes with LHAs can be achieved rapidly using PCR based fragment fusion approaches (Zhao et al. 2019). However, generation of gene tagging or promoter replacement cassettes where there is limited flexibility at the integration site, or point mutation cassettes, where four or more DNA fragments need to be combined becomes difficult and laborious.

Genome editing techniques utilising CRISPR-Cas9 mediated transformations have been applied in filamentous fungi to overcome some of these issues (Barrangou et al., 2007, Fuller et al., 2015, Liu et al., 2015, Matsu-Ura et al., 2015, Nodvig et al., 2015). The utility of a CRISPR-Cas9 system was first exemplified in *A. fumigatus* by performing locus directed, randomised mutagenesis at the site of the DHN-melanin biosynthesis gene (*pksP*) (Fuller et al. 2015). Although successful, mutagenesis was dependent on constitutive *in vivo* expression of Cas9, which can be detrimental to cell viability and function, (Haapaniemi et al., 2018, Kim et al., 2018, Kosicki et al., 2018) and the co-expression of a selectable marker. Following this study, the first micro-homology arm (MHA) mediated CRISPR-Cas9 gene replacement system was developed. This marked a significant advance over standard homology based gene replacement and tagging systems in *A. fumigatus*, as MHAs (28 bp) could be used to target insertion sites. However, there was still a requirement for the Cas9 enzyme to be expressed *in vivo*, albeit under the control of a regulatable promoter. Additionally, two separate selectable markers were required for the procedure (Zhang et al. 2016). This system was used to insert a SNP in the *pksP* gene, a GFP tag at the N-terminus of calcineurin (Zhang et al. 2016) and been used for promoter replacement (Rybak et al. 2019).

Recently, an *in vitro* assembled CRISPR-Cas9-gRNA (IVACC) transformation system that utilised micro-homology repair templates was developed (Al Abdallah et al. 2017). This methodology is highly efficient, rapid and does not require *in vivo* expression of the Cas9 nuclease and gRNA. This limits the possibility of off-target effects caused by long term expression of *cas9* (Al Abdallah et al. 2018). Here we present further exemplification of this IVACC mediated transformation methodology and show it can be widely applied for various genetic manipulations. Specifically, we demonstrate that gene insertion, protein fusion and point mutations are generated with such high efficiency that the technique can be carried out without the need of a selection marker, therefore bypassing, in many instances, the need to generate complex constructs.

## 2 Materials and methods

### 2.1 Strains, plasmids, gRNA and repair template generation

*A. fumigatus* strains wild-type strains Af293 and CEA10 along with MFIG001, a member of the CEA10 laboratory lineage lacking a functional *ku80* gene, were used as the parental isolates for the transformations (Bertuzzi et al., 2020, Furukawa et al., 2020). Where the hygromycin resistance cassette was used as a selectable marker, it was amplified from the pAN7.1 plasmid (available from the Fungal Genetics Stock Centre) using primers detailed in the results section and Table S1.

Target specific crRNAs as well as oligos to prepare a repair template were designed using a web-based guide RNA designing tool EuPaGDT (Peng and Tarleton 2015). The genome sequence of *A. fumigatus* A1163, which was downloaded from the CADRE genomic database, was manually uploaded to EuPaGDT, and the program was executed with default setting to design gRNAs to the *aft4* (Hey 2007)*, pacC* and *srbA* loci. As a result, several candidate crRNAs were obtained. Those crRNAs closest to the target integration sites with the highest QC scores were manually selected for the transformation experiments (Table S1).

**Supplementary figure 1:**
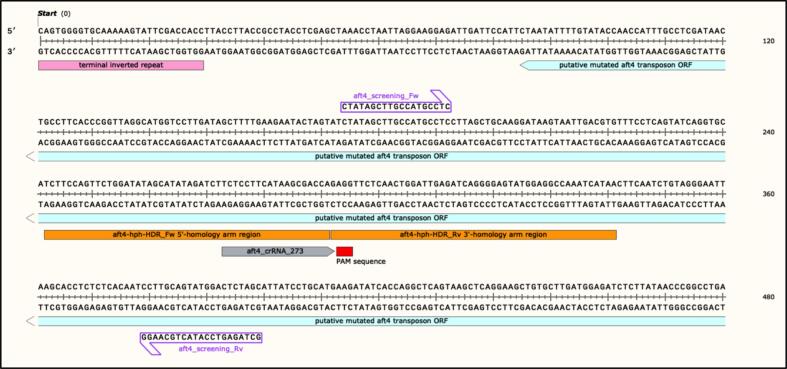
Genome editing of the *aft4* locus. crRNA and the primers used for genome editing of the *aft4* locus are shown. PAM sequence to the corresponding crRNA is indicated by red. Nucleotide sequences of the crRNA and the primers are also available in Table S1.

Homology directed repair (HDR) templates were amplified using primers that incorporated 50-bp microhomology arms (MHAs) (see Fig. 2, Fig. 3, Supplementary figure 1, Supplementary figure 3, Supplementary figure 6, Supplementary figure 7 and Table S1). The *e*g*fp-* or the 3xFLAG-containing repair templates were amplified from pUgfp-pacCTF (the *egfp* fragment was originally sourced from pCH008 (Helmschrott et al. 2013)) or pUpacC-3xFLAG (a plasmid containing a codon optimized 3xFLAG tag-*pacC* fusion gene) by PCR with a corresponding pair of primers listed in Table S1 using Phusion Flash Master Mix (Thermo Fisher Scientific). The amplified MHA templates were gel purified (Qiagen PCR purification kit) and used for transformation. The guide RNAs and primers used for the CRISPR-Cas9 mediated transformation are listed in Table S1.

### 2.2 Transformation and validation of transformants

Our CRISPR-Cas9 transformation protocol is based upon methodology from Zhao et al., while RNP assembly is based upon Al Abdallah et al (Al Abdallah et al., 2017, Zhao et al., 2019). Conidia were inoculated in liquid *Aspergillus* Complete Medium (ACM) at a concentration of 1 × 10^6^ conidia/mL and cultured for 16 h at 37 °C with shaking at 120 rpm (Cove 1966). Mycelia were harvested through filtration with Miracloth and resuspended in ACM with protoplasting buffer (10 g Vinotaste®Pro [Lamoth-Abiet] in 100 mL 1 M KCl + 0.1 M Citric Acid, filter sterilised through 0.22 µm PVDF-filter and added to 100 mL ACM liquid medium). This was incubated for 4 h at 37 °C with shaking at 120 rpm. Protoplasts were harvested through filtration with Miracloth and centrifuging for 10 min at 2,000 × *g*. Protoplasts were washed 3 times in 0.6 M KCl and resuspended in 0.6 M KCl + 50 mM CaCl_2_. Protoplasts were counted using a haemocytometer and diluted to 1 × 10^6^ protoplasts/mL in 0.6 M KCl + 50 mM CaCl_2_. 50 µL of this protoplast solution was used per transformation.

RNP complexes were assembled *in vitro* using purified Alt-R® *Streptococcus pyogenes* Cas9 V3 protein, a 67 mer Alt-R® CRISPR-Cas9 tracrRNA (100 µM stock) and locus specific Alt-R® CRISPR-Cas9 crRNA (100 µM stock) (Integrated DNA Technologies). Alt-R® S.p. Cas9 Nuclease contains a nuclear localisation sequence (NLS). Equimolar concentrations of tracrRNA and crRNA were resuspended in Nuclease-Free IDTE Buffer, following manufacturers’ protocol (Integrated DNA Technologies). This was heated to 95 °C for 5 min and cooled to room temperature gradually in a thermal cycler for 10 min (0.1 °C/s). Hybridised crRNA and tracrRNA were mixed with Alt-R® S.p. Cas9 protein (1 µg/µL) in Cas9 working buffer (20 mM HEPES; 150 mM KCl, pH 7.5) and incubated at room temperature for 5 min.

The RNP complexes (33 µM RNA duplex with 1.5 µg Cas9 in Cas9 working buffer, total volume of 26.5 µL) were mixed with the purified protoplasts (5 × 10^4^ protoplasts in 50 µL), repair template (0.5–1 µg of dsDNA repair template or 200 pmol of ssDNA), and PEG-CaCl_2_ buffer (60% wt/vol PEG3350, 50 mM CaCl_2_, 450 mM Tris-HCl, pH 7.5) and incubated on ice for 50 min. 1 mL of PEG-CaCl_2_ was then added to the mixture and incubated at room temperature for 25 min. Either, the entire transformation mixture was plated over 2 YPS (2% yeast extract, 5 mM Tris base, 0.5% peptone, 2% glucose, 1 M sorbitol, adjusted to pH 6.0) plates containing either hygromycin (Apollo Scientific, 100 μg/mL) or no selection and incubated at room temperature for 24 h, or the transformation mix was diluted to 3x10^3^ per mL in 0.6 M KCl + 50 mM CaCl2 and c. 300 protoplasts were plated. Subsequently, plates were incubated at 37 °C for 3 days. Transformants were purified twice by streaking on ACM. For introduction of the *pyrG* point mutation, Aspergillus Minimal Medium (AMM) supplemented with 5 mM uridine and 5 mM uracil was used as a non-selective medium (Pontecorvo et al. 1953). Spores from purified colonies were harvested in PBS (Phosphate Buffered Saline, Sigma-Aldrich) + 0.1% Tween 20 (Sigma-Aldrich) for downstream analysis.

DNA was extracted from *A. fumigatus* spores via Cetyl Trimethyl Ammonium Bromide (CTAB) extraction (Fraczek et al. 2013). PCR was performed to validate successful incorporation of cassettes using Phusion Flash Master Mix (Thermo Fisher Scientific) and relevant primer pairs (Table S1). PCR products were assessed by gel electrophoresis. Sanger sequencing was performed for three independent isolates by Eurofins Genomics, Genewiz or the University of Manchester Genomic Core Facility. The genomic regions subjected to the sequencing analysis are indicated in Figs. S3, S6 and S7, and the primers used for the analysis are listed in Table S1. Transformation efficiency was calculated by dividing the number of PCR positive strains from the total number of strains assessed.

### 2.3 Fluorescent microscopy

2000 spores of *A. fumigatus* SrbA-GFP and GFP-PacC were grown for 16 h at 37 °C in 200 μL Watch Minimal Media (WMM, pH 5.5) in an 8 well chamber (Ibidi) (Penalva 2005) after which time the media was replaced with WMM + 0.25 mg/L itraconazole (Sigma) or WMM (pH 8.0), respectively for the SrbA-GFP and GFP-PacC strains. Fluorescent and bright field live-cell imaging were performed using a Leica TCS SP8 confocal laser scanning microscope equipped with hybrid GaAsP (HyD) detectors and a 63 × water immersion objective. Argon laser 488 nm was used for fluorescence excitation. Confocal microscopy images were analysed and processed with Imaris 8.0 developed by Bitplane (Zurich, Switzerland).

## 3 Results

### 3.1 High efficiency transformation using an *in vitro* CRISPR-Cas9 system reveals the potential for marker-less genome editing of *Aspergillus fumigatus*

In previous studies that utilise IVACC mediated transformation, two different gRNAs, flanking the region to be replaced, were used to generate double stand breaks and promote incorporation of a gene replacement cassette in the *A. fumigatus* genome (Fuller et al., 2015, Al Abdallah et al., 2017). Although single gRNA mediated gene replacement has previously been reported in *Aspergillus nidulans, Aspergillus niger* and *Aspergillus oryzae* (Nodvig et al. 2018), it was unclear if single gRNAs would be compatible with IVACC to facilitate gene integration.

**Fig. 1:**
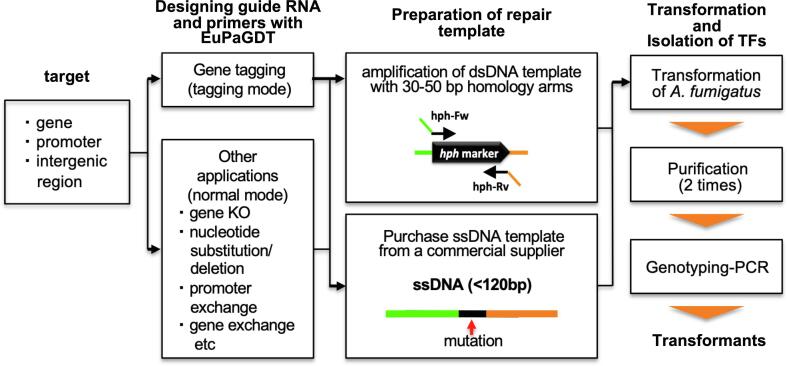
**Workflow of CRISPR-Cas9 mediated transformation in *A. fumigatus*.** The target sequence was used as input for EuPaGDT to design the gRNA. This tool includes tagging mode for epitope tagging and normal mode for other applications. In addition, designing the gRNA using EuPaGDT returns the required sequence of the homology arms. The repair template can either be designed to be amplified or purchased as an oligo.

We adopted an experimental pipeline (Fig. 1) by combining a web-based gRNA designing software EuPaGDT (http://grna.ctegd.uga.edu/) (Peng and Tarleton 2015) and the IVACC mediated transformation system (Al Abdallah et al. 2017) to assess the efficiency of single gRNA mediated insertion of a 2.7-kb hygromycin resistance marker (*hph*) with 50 bp homology arms into the *A. fumigatus* genome (Fig. S1). To this end, a guide RNA was designed to target *aft4*, a locus that appears to encode a non-functional transposable element due to a non-sense mutation within the putative transposase-encoding gene (Hey 2007). Detailed molecular characterization of this locus will be described elsewhere (Furukawa *et al*. in preparation).

**Fig. 2:**
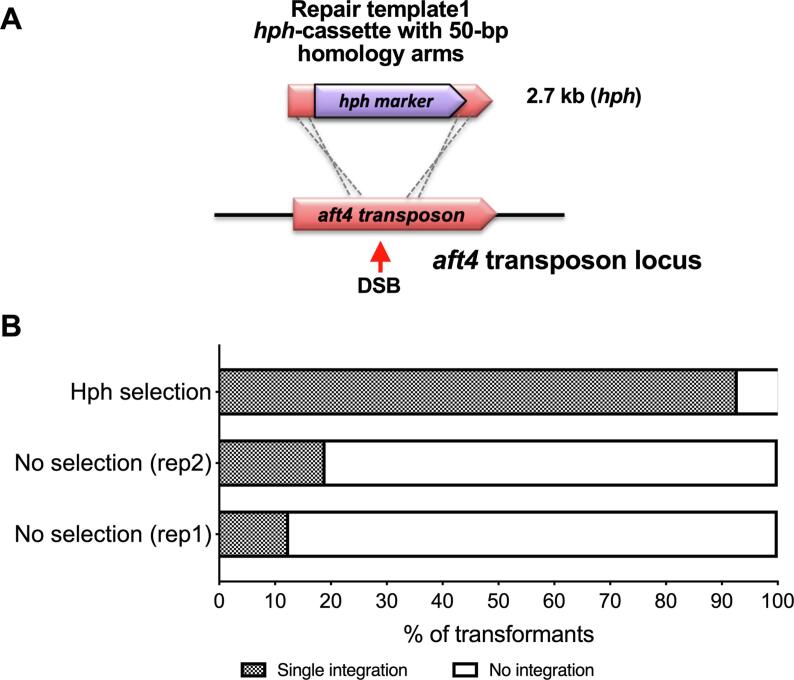
**CRISPR-Cas9 transformation using a selective and non-selective conditions A.** Schematic representation of CRISPR-Cas9 mediated insertion of the hygromycin selection marker into the *aft4* locus*.***B.** Efficiency of integration of DNA into the *A. fumigatus* MFIG001 genome either with or without hygromycin selection after transformation. Under selective conditions efficiency is up to 90% of all transformants, while under non-selective conditions, efficiency is 10–20%.

**Supplementary figure 2:**
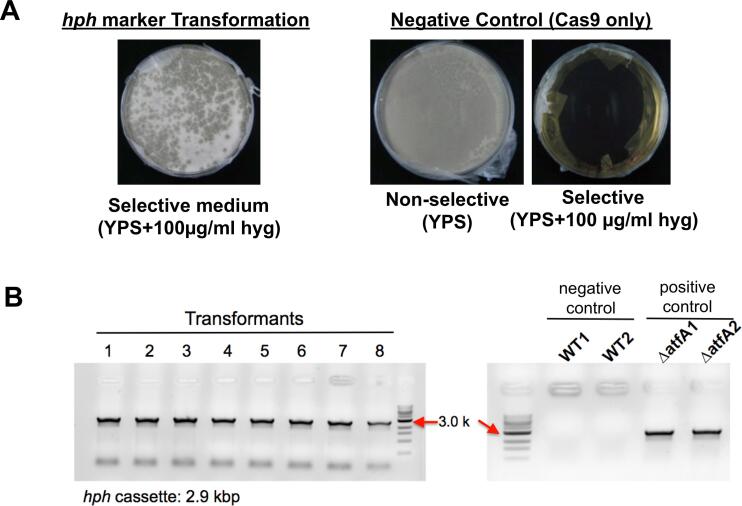
Screening of *hph* insertional mutants at the *aft4* transposon locus A. gRNA was designed to cause a double stranded break (DSB) in the *atf4* transposon locus, which is a defective transposon. The *hph* marker was amplified with 50bp homology arms towards the flanking region of the DSB, to guide integration towards this site. After CRISPR transformation this resulted in several 100 transformants on a YPS+hygromycin petridish, while the negative control under selective media was clear. B. Gel electrophoresis of PCR amplified hygromycin resistance cassettes using aft4_screening_fw and aft4_screening_fw primer for transformants screened under selective pressure. A 2.9kb product was expected. Positive controls containing genomic DNA from a Δaft4 mutant was included, where *aft4* was replaced with the hygromycin selection marker by heterologous recombination. Design of the PCR can be found in Figure S1.

In our initial experiment gRNA^aft4^-Cas9 complex mediated transformation of the *hph* cassette into MFIG001 protoplasts yielded 272 hygromycin resistant colonies from c.300 protoplasts, indicating that the *hph* cassette had been incorporated into the nuclei of around 91% of protoplasts. 95 candidate transformants were single colony purified two times under hygromycin selection and integration of the *hph* cassette at the *aft4* locus was assessed by PCR (Figure Supplementary figure 2Fig. S2). This revealed a remarkably high efficiency of gene integration (93%) using a single gRNA (Fig. 2).

As the frequency of gene integration in our pilot experiment was so high, we investigated the possibility of non-selection marker mediated transformation of *A. fumigatus*. Again, we performed a gRNA^aft4^-Cas9 complex mediated transformation of MFIG001 protoplasts using the same mutagenesis cassette. However, on this occasion, the transformation mix was plated on media lacking hygromycin. Growing colonies were colony-purified twice under non-selective conditions. Of the candidate transformants assessed by PCR from 2 independent experiments (n = 95 from each), 19% and 12.5% of transformants resulted in a positive signal for integration of the *hph* cassette-containing repair template (Fig. 2).

### 3.2 Development of a marker-free epitope-tagging method using the IVACC system

**Supplementary figure 3:**
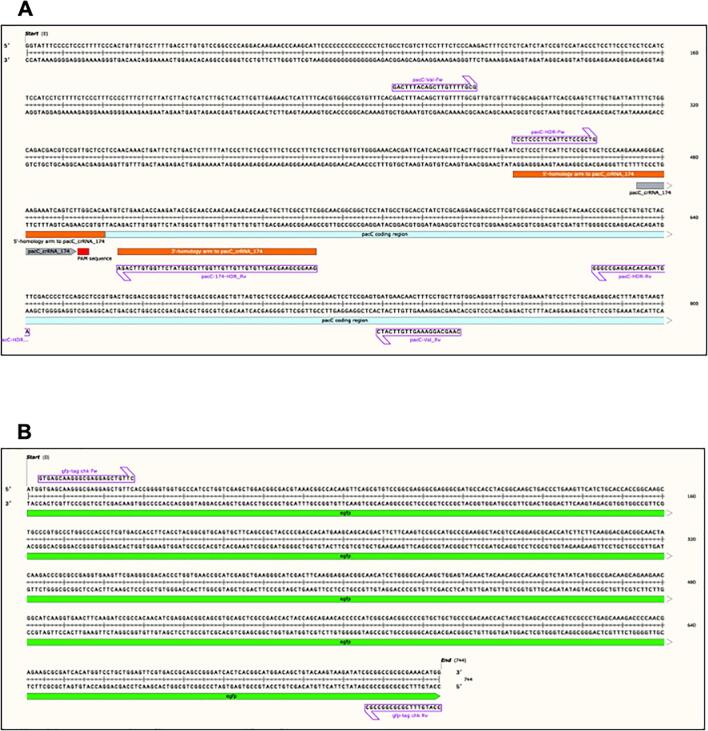
Genome editing of the *pacC* locus. A. genome editing of the N-terminal region of *pacC*. B. The nucleotide sequence of the *egfp* encoding gene. All the crRNA and the primers used for the *gfp*-tagging of *pacC* study are shown. PAM sequence to the corresponding crRNA is indicated by red. Nucleotide sequence of the crRNAs and the primers is also available in Table S1.

**Supplementary figure 4:**
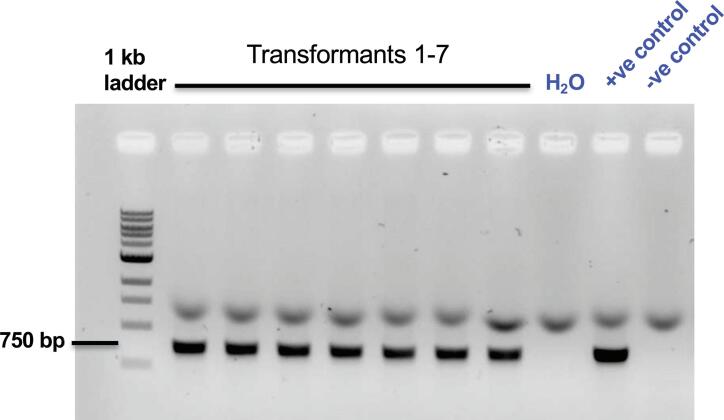
PCR screening of *gfp-pacC* transformants. Gel electrophoresis of PCR amplified (n = 7) GFP with primers GFP_tag_chk_FW and GFP_tag_chk_RV from screened GFP-*pacC* transformants (n = 90). A product of 750 bp was expected. Negative controls contained A1160 genomic DNA and H_2_O as template DNA. Positive controls containing the pUgfp-pacCTF plasmid as template DNA were included. Design of the PCR can be found in Figure S3.

Chimeric proteins are often used to facilitate our understanding of a number of aspects of protein function such as protein localisation and identification of interacting partners. The generation of protein fusion cassettes can however be a time-consuming process, requiring integration of multiple components. Our ability to perform directed gene replacement without the need for selection prompted us to assess if we would be able to introduce epitope-tags to genes using a PCR amplified *egfp* cassette with MHA without incorporating a selectable marker. The well characterized pH-responsive transcription factor PacC was selected as a target for our study (Bertuzzi et al. 2014). A cassette containing the *egfp* gene and 50-bp MHA designed to direct insertion at the *pacC* start codon was PCR amplified and introduced into MFIG001 using IVACC mediated transformation (Fig. S3). We selected 95 candidate strains that were recovered on, and subsequently purified on a non-selective medium (Sabouraud agar) as before. Integration of the *egfp* tag was assessed by PCR (Figure Supplementary Fig. S4). Consistent with the results of the insertion of the *hph* marker, we successfully obtained transformants, which showed integration of *egfp*-tag at the desired locus for 13/95 (14%) of strains. Precise integration of *egfp* at the start codon of the *pacC* gene and absence of any other mutations at this site was confirmed by Sanger sequencing of three isolates.

**Fig. 3:**
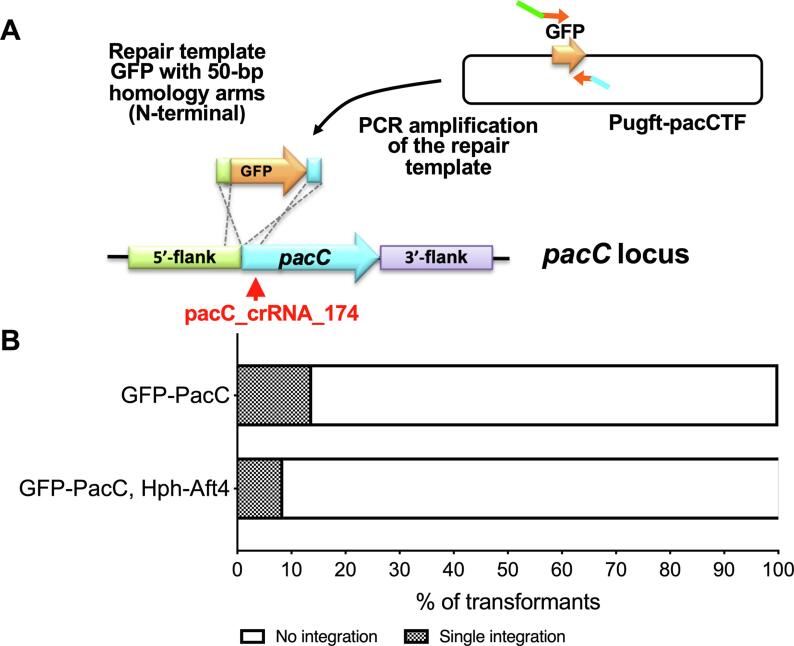
**Epitope tagging using non-selective CRISPR-Cas9 transformation and co-transformation with a selectable marker A.** Schematic of epitope tagging for *egfp*-*pacC*. A repair template containing the *egfp* gene and 50-bp homology arms flanking the *pacC* start codon was amplified from pUgfp-pacCTF and introduced by CRISPR-mediated transformation using pacC_crRNA_174 as crRNA **B.** Efficiency of N-terminal GFP integration to the *pacC* locus with and without cotransformation of the hygromycin selection marker towards the *aft4* locus.

**Supplementary figure 5:**
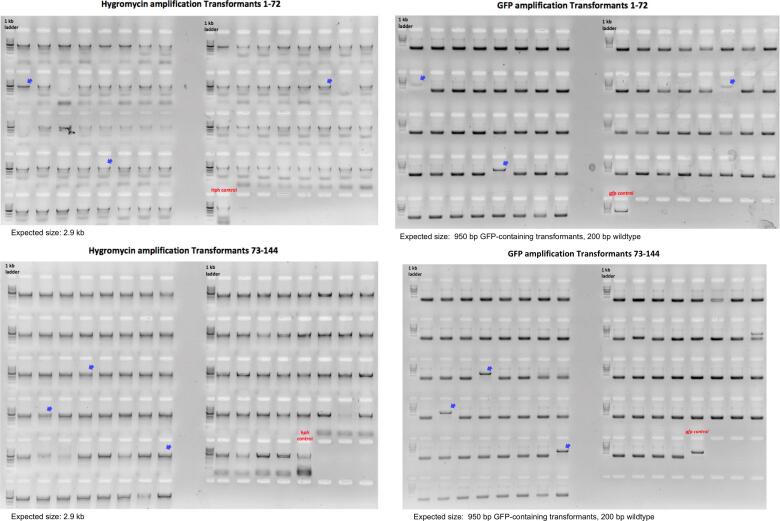
PCR screening of *gfp-pacC-aft4-hph* co-transformants. Gel electrophoresis (1% agarose) of PCR amplified GFP, using primers pacC_HDR_fw and pacC_HDR_Rv, from CRISPR transformed GFP-*pacC* co-transformed with *aft4-hph*. Blue arrows represent positive signal detecting GFP and hygromycin (*hph*) amplicons. Design of the PCR can be found in Figure S3.

In an attempt to improve the efficiency of integration of the *egfp*-tag, we investigated a co-transformation approach, in which the *pacC*-targeted *egfp*-tag was introduced together with the *aft4* targeted *hph* marker. The transformants were selected on media containing hygromycin and analyzed by PCR as described above (Figure Supplementary Fig. S5). No clear improvements were observed, as only 8.5% of the *hph* positive transformants showed co-insertion of the *egfp*-tag at the *pacC* locus (Fig. 3).

**Fig. 4:**
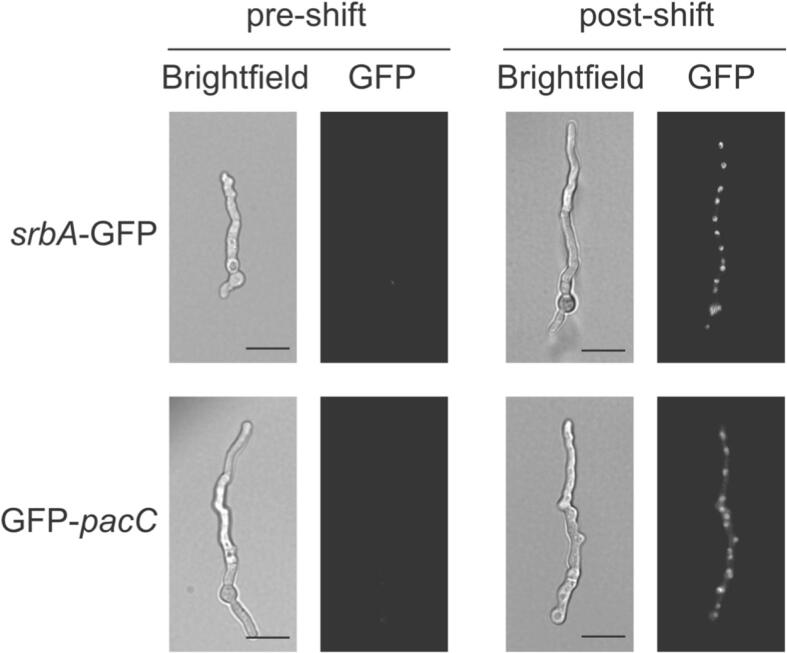
**Localisation of epitope tagged *srbA* and *pacC*.** Fluorescence microscopy of the *A. fumigatus* MFIG001 GFP-SrbA and GFP-PacC after itraconazole or pH shift, respectively (scalebar equals 10 μm).

**Supplementary figure 6:**
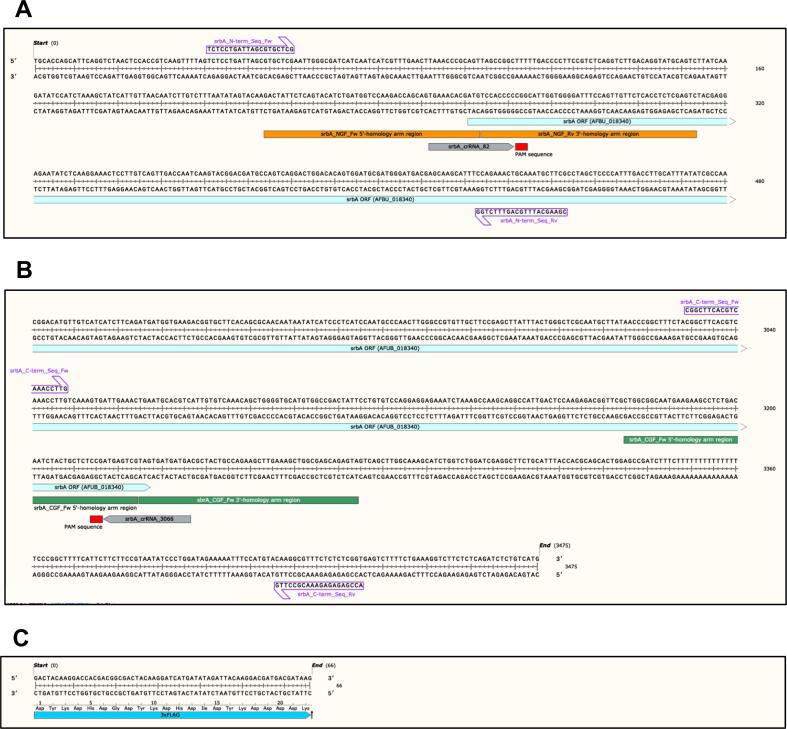
Genome editing of the *srbA* locus. A. genome editing of the N-terminal region of *srbA*. B. genome editing of the C-terminal region of *srbA*. C. The nucleotide sequence of the codon optimized 3xFLAG tag used for the *srbA* tagging study. All the crRNAs and the primers used for the *srbA* tagging experiments are shown. PAM sequence to the corresponding crRNAs are indicated by red. Nucleotide sequence of the crRNAs and the primers is also available in Table S1.

To ensure that CRISPR mediated tagging was not locus- and tag-dependent, we also sought to fuse the sterol regulatory element binding protein SrbA with eGFP- and 3XFLAG at both its N and C termini (Willger et al. 2012). Four different MHA cassettes with 50-bp homology arms (eGFP-C-tag, e-GFP-N-tag, 3xFLAG-C-tag and 3xFLAG-N-tag) were generated by PCR and used to transform *A. fumigatus* MFIG001 via our selection-free method (Fig. S6). For each cassette we isolated and purified 10 different transformants on a non-selective medium (Sabouraud agar), and examined integration of each tag by PCR. In agreement with the frequency of successful non-selection IVACC transformation, we obtained two GFP C-terminal SrbA tagged strains, two N-terminal 3xFLAG tagged SrbA strains and one C-terminal 3xFLAG tagged SrbA strain. No N-terminal eGFP tagged SrbA strains were obtained. We were able to confirm precise integration of the epitope-tag cassette without introducing any other mutation at the target site by Sanger sequencing (Sequenced region shown in Fig. S6). To evaluate if the eGFP tagged proteins were correctly expressed and localized we assessed the GFP-PacC and SrbA-GFP modified strains using confocal microscopy. In keeping with the role of PacC in alkaline mediated regulation and SrbA in the regulation of ergosterol biosynthesis (Willger et al., 2008, Bertuzzi et al., 2014, Vaknin et al., 2016) we confirmed condition dependent nuclear localisation of GFP-PacC after alkaline shift and of SrbA-GFP after itraconazole mediated ergosterol depletion (Fig. 4).

### 3.3 Direct introduction of a point mutation using the CRISPR-Cas9 system

Generation of targeted point mutations in *A. fumigatus* is complicated by the need to incorporate a selectable marker near the site of the modification that is being made. It is therefore unclear if any change in the phenotype of a modified strain is caused by the point mutation or as a result of changes in the genomic context around the mutation site. Our non-selective CRISPR-mediated transformation process has the potential to both reduce the need for time-consuming and expensive construction of site-directed mutagenesis vectors, and also permit precise nucleotide level modification (Table S2).

**Supplementary figure 7:**
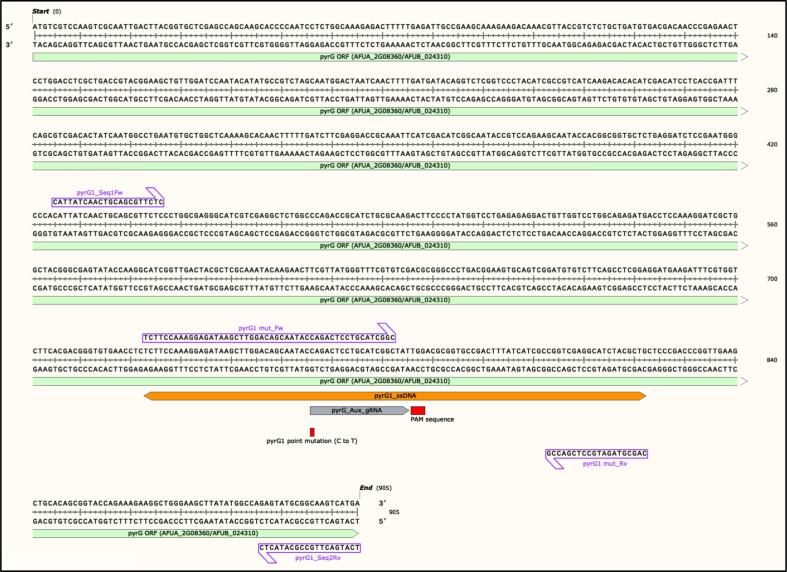
Genome editing of the *pyrG* locus. Genome editing of the *pyrG* locus. The crRNA, the primers, and the single strand DNA repair template used for the *pyrG* mutation study are shown. Nucleotide sequence of the crRNAs and the primers is also available in Supplementary Table 1.

Here, we explored our ability to generate a targeted point mutation (C to T) at base position 1413 of *pyrG* gene (to generate a stop codon as exemplified in the AFpyrG1 strain (Weidner et al., 1998), herein defined as the *pyrG1* mutation). We performed the mutagenesis in two *A. fumigatus* wild-type strains, Af293 and CEA10. Unlike strain MFIG001, both of these strains have their NHEJ pathway intact making successful homologous integration of endogenous DNA less efficient. We assessed if a 100 bp single stranded DNA oligonucleotide could be used for generating a targeted point mutation (Fig. S7).

**Fig. 5:**
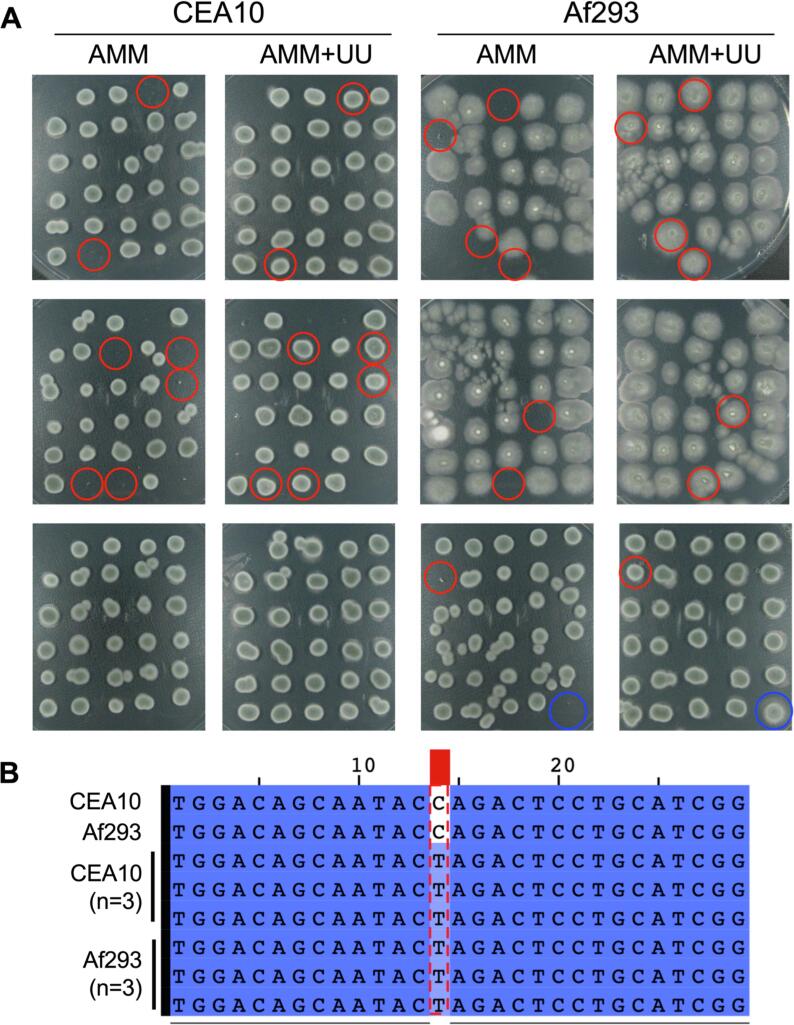
**Generation of a point mutation in the *pyrG* locus via selection free CRISPR-mediated transformation. A.** Spot test of 85 and 88 transformants of *A. fumigatus* CEA10 and Af293, respectively, after selection free ssDNA mediated CRISPR transformation of the *pyrG* locus. Transformants were twice purified and 100 spores were spotted on Aspergillus Minimal Medium (AMM) or AMM supplemented with 10 mM uracil and uridine (AMM + UU) and grown for 48 h. The *A. fumigatus* A1160 control is circled in blue and strains showing a phenotype consistent with loss of PyrG function are circled red. **B.** Sequencing result from 6 transformants (3 CEA10 and 3 Af293) obtained via non-selective transformation that were positive via spot test phenotyping. The targeted point mutation is highlighted in red. (For interpretation of the references to colour in this figure legend, the reader is referred to the web version of this article.)

We performed the IVACC mediated transformation with ssDNA, and colony purified 85 and 88 viable colonies from CEA10 and Af293, respectively. A spot test on AMM and AMM supplemented with uridine and uracil was performed to assess the phenotypes of the strains (Fig. 5A). Isolates showing growth on supplemented media, while being unable to grow on un-supplemented media were considered successful transformants. In total, 8.2% (7/85 transformants) and 8% (7/88 transformants) showed the phenotype consistent with loss of PyrG function for CEA10 and Af293, respectively. A 481 bp fragment was amplified and sequenced from the *pyrG* gene of 3 transformants from each parental isolate. The targeted point mutation was present in all 6 transformants and no off-target mutations were detected in the region assessed (Fig. 5B).

## 4 Discussion

CRISPR-Cas9 systems have been revolutionary for our ability to perform genetic manipulation in filamentous fungi, most notably because they allow us to dispense with the need to create constructs that have long homology arms of more than 500 bp. The first systems to be employed in filamentous fungi used *in vivo* expressed Cas9 endonuclease. In these systems, expression of Cas9 is under the control of constitutive or highly expressed inducible promoters resulting in the production of high levels of Cas9 protein. Evidence from *S. cerevisiae* and *Magnaporthe oryzae* suggests overexpression of Cas9 is toxic to cells resulting in reduction of viability, in line with observations in human cell lines (DiCarlo et al., 2013, Morgens et al., 2017, Foster et al., 2018). To overcome Cas9 mediated toxicity, a recent iteration of CRISPR based transformation technology, first employed in mammalian cells and then adapted for *A. fumigatus,* uses *in vitro* assembled RNP complexes and has been shown to be highly efficient and does not result in detectable levels of off-target mutations (Kim et al., 2014, Liang et al., 2015, Al Abdallah et al., 2017, Al Abdallah et al., 2018). This system is highly versatile and requires very little up-front investment (reagents for each RNP complex cost c. £50, Table S2) and there is no need to develop expression constructs for either Cas9 or gRNAs. We therefore chose to assess the performance of this system in our laboratory. In agreement with the work published by Al Abdallah et al, we found that transformation rates using this system were very high (Al Abdallah et al. 2017). By titrating the number of protoplasts we used in our transformation experiments we discovered that almost all cells (>90%) were modified during the transformation procedure. This clearly suggested that the selectable marker was dispensable for the transformation process. Using a selection free approach, over 10% of strains purified from IVACC transformed colonies were shown to contain the transforming DNA. Although we did not investigate the discrepancy between the efficiency we observed in the selective and non-selective methods, it is likely to be linked to our use of protoplasts, which are typically multi-nucleate and hence upon transformation would result in the generation of heterokaryotic progeny. Importantly we have used the IVACC mediated transformation system to exemplify how epitope tagged fusion proteins, and point mutations can be generated efficiently without selection.

Marker-free transformation marks a significant advance in our ability to perform genetic manipulation in *A. fumigatus* as we now have the ability to generate precise mutations while keeping the flanking regions of the target gene unedited. Insertion of a selectable marker, driven by a strong promoter can have an effect on expression of neighbouring genes or the target itself and can result in uncoupling of the coding sequence from *cis*-regulatory elements such as transcription factor binding sites and regulatory 3′ UTRs (Morozov et al., 2000, Gsaller et al., 2016). CRISPR facilitated ‘marker-free’ selection systems have previously been described for use in *A. oryzae, A. nidulans* and *A. niger* (Nodvig et al. 2018), however these systems, necessitate cloning of gRNAs into shuttle-vectors and hence are more time consuming than the IVACC system described herein. The methodologies outlined by Nodvig et al used strains that lack orotidine 5′-phosphate decarboxylase (PyrG) activity to take advantage of bi-directional selection to facilitate maintenance and ultimate removal of the AMA1 extra-chromosomal plasmid from which *cas9* was expressed, hence the selectable marker was not retained in the modified strain. As *A. fumigatus* strains that lack *pyrG* are avirulent (D'Enfert et al. 1996), direct implementation of this system is of limited use when exploring factors governing pathogenicity. However, a recent exemplification of a similar system in *Aspergillus carbonarius* highlights that an AMA1 cassette carrying a *cas9* expression cassette and positive selectable marker (*hph*) is readily lost through multiple passages on non-selective media (Weyda et al. 2017) suggesting that modified strains lacking a selectable marker can be generated in this way, albeit using a more complex process.

It is particularly noteworthy that we were able to generate specific point mutations using our selection free approach even in wild-type strains that are NHEJ proficient indicating that we will be able to perform genetic manipulation in a wide range of clinical and environmental isolates of *A. fumigatus*.

In summary we have generated and optimised a CRISPR-Cas9 transformation methodology without the need of dominant selection markers. This transformation methodology can be used for the generation of gene deletion mutants, point mutants and protein fusions at relatively high efficiency (approaching 20%). This efficiency appears to be independent of the targeted locus or epitope used. These methodologies allow targeted genetic manipulations that overcome the need for time consuming and complex cassette construction. They also replace the need to use dominant selection markers with promoters that drive high levels of expression and have the potential to interfere with the outcome of experiments (Yau and Stewart 2013). Overall, this methodology may facilitate more efficient genome editing and reduce off target effects caused by introduction of large constructs in *A. fumigatus* and other fungi.

## CRediT authorship contribution statement

**Norman van Rhijn:** Conceptualization, Investigation, Formal analysis, Writing - original draft, Visualization. **Takanori Furukawa:** Conceptualization, Investigation, Formal analysis, Writing - original draft. **Can Zhao:** Investigation. **Bethany L. McCann:** Investigation. **Elaine Bignell:** Conceptualization, Supervision, Funding acquisition. **Michael J. Bromley:** Conceptualization, Methodology, Supervision, Writing - original draft, Writing - review & editing, Funding acquisition.

## Declaration of Competing Interest

MJB is a former consultant to Synlab GmbH, is the director and shareholder of Syngenics Limited and is a substantive shareholder in PiQ Laboratories Ltd. The remaining authors declare no competing interests.
